# Angiotensin II Receptor Blockers but Not Angiotensin-Converting Enzyme Inhibitors Are Associated With a Reduced Risk of Acute Kidney Injury After Major Surgery

**DOI:** 10.3389/fphar.2021.662301

**Published:** 2021-04-23

**Authors:** Shao-Yu Yang, Tao-Min Huang, Tai-Shuan Lai, Nai-Kuan Chou, Chun-Hao Tsao, Yi-Ping Huang, Shuei-Liong Lin, Yung-Ming Chen, Vin-Cent Wu, NSARF study group

**Affiliations:** ^1^ Department of Internal Medicine, National Taiwan University Hospital and College of Medicine, Taipei, Taiwan; ^2^ Surgery, NSARF, National Taiwan University Hospital Study Group on Acute Renal Failure, Taipei, Taiwan

**Keywords:** angiotensin-converting-enzyme inhibitors, angiotensin receptor blockers, postoperative, acute kidney injury, mortality

## Abstract

**Objective:** We investigated the respective effects of preoperative angiotensin-converting-enzyme inhibitors (ACEIs) and angiotensin receptor blockers (ARBs) on the incidence of postoperative acute kidney injury (AKI) and mortality.

**Methods:** In this nested case-control study, we enrolled 20,276 patients who received major surgery. We collected their baseline demographic data, comorbidities and prescribed medication, the outcomes of postoperative AKI and mortality. AKI was defined by the criteria suggested by KDIGO (Kidney disease: Improving Global Outcome). Logistic regression was used to assess the impact of exposure to ACEIs or ARBs.

**Results:** Compared with patients without ACEI/ARB, patient who received ARBs had a significantly lower risk for postoperative AKI (adjusted odds ratio (OR) 0.82, *p* = 0.007). However, ACEI users had a higher risk for postoperative AKI than ARB users (OR 1.30, *p* = 0.027), whereas the risk for postoperative AKI was not significantly different between the ACEI users and patients without ACEI/ARB (OR 1.07, *p* = 0.49). Compared with patients without ACEI/ARB, both ACEI and ARB users were associated with a reduced risk of long-term all-cause mortality following surgery (OR 0.47, *p* = 0.002 and 0.60, *p* < 0.001 in ACEI and ARB users, respectively), without increasing the risk of hyperkalemia during the index hospitalization (*p* = 0.20). The risk of long-term all-cause mortality following surgery in ACEIs and ARBs users did not differ significantly (OR 0.74, *p* = 0.27). Furthermore, the higher the defined daily dose of ARB, the better the protection against AKI provided.

**Conclusion:** Our study revealed that preoperative use of ARBs was associated with reduced postoperative AKI, which is better in high quantity, whereas preoperative use of ACEIs or ARBs were both associated with reduced mortality and did not increase the risk of hyperkalemia.

## Introduction

Angiotensin-converting-enzyme inhibitors and angiotensin receptor blockers are both used frequently in the treatment of patients with hypertension, congestive heart failure, or chronic kidney disease. However, out of concern for their possible effects on kidney function deterioration, it has been suggested that ACEIs/ARBs should be withheld prior to or during some clinical scenarios, including the perioperative period (Che et al., 2011).

Postoperative acute kidney injury remains common, even though perioperative care has improved greatly; particularly in high-risk patients, the incidence of AKI may be as high as 20–40% (Bauerle et al., 2011). Postoperative AKI is noteworthy because even minor increases in creatinine levels will increase the risk of death and the length of hospitalization (Kork et al., 2015). However, previous studies exploring the impacts of preoperative use of ACEIs/ARBs on postoperative AKI provided conflicting data. Though a retrospective cohort study of 1,358 adults revealed that preoperative use of ACEIs/ARBs associated with an increased risk for AKI after cardiovascular surgery (Arora et al., 2008), a newer prospective cohort study of 1,594 adults revealed that preoperative ACEIs/ARBs usage was associated with functional but not structural AKI after cardiac surgery (Coca et al., 2013). However, some studies showed that ACEIs/ARBs were not associated with postoperative AKI (Tagawa et al., 2015; Bonavia et al., 2019), and a study suggested that preoperative ACEIs/ARBs are associated with reduced postoperative AKI requiring dialysis and even reduced mortality (Shah et al., 2014). The risk and benefit of perioperative use of ACEIs/ARBs remained uncertain.

Further, ACEIs and ARBs are basically distinct because they inhibit different parts of the renin-angiotensin-aldosterone system (RAS) though many of their pharmacologic effects are similar. Previous studies focusing on the effects of these two kinds of agents on postoperative AKI rarely explored their respective roles. Therefore, in the present study, we tried to explore whether the use of ACEIs or ARBs before major surgery associates with an increased incidence of postoperative AKI and mortality, respectively. In addition, we tried to find out the association between the dose and the effects in ACEIs and ARBs.

## Methods

### Patients

The retrospective study enrolled patients between 20 and 75 years old who received major surgery since May 2013 to December 2017 in the National Taiwan University Hospital (NTUH), a tertiary-care referral center and two regional rural hospitals, the Hsin-Chu and Yun-Lin Branches of the NTUH. Surgical procedures were considered major if the length of the intensive care unit stay for patients in a given diagnosis-related group exceeded two days (Lindenauer et al., 2005). Major surgery procedures were further classified into cardiothoracic, esophagus, intestine, and liver as the main diagnostic codes (Lin et al., 2018).

The detailed clinical and laboratory information of these patients was obtained from the Integrated Medical database (NTUH-iMD). Patients who received dialysis within 90 days before surgery were excluded. The record of usage of ACEIs or ARBs within 90 days before surgery were collected to determine the group classification. Patients who used ACEIs and ARBs simultaneously within 90 days before surgery were excluded to avoid misclassification, and the others were classified into three groups: those who received ACEIs, those who received ARBs, and those who received neither ACEI nor ARB within 90 days before surgery. The baseline demographic data, comorbidities, and the medication prescribed to these patients were collected. The estimated glomerular filtration rate (eGFR) was calculated with the Modification of Diet in Renal disease Study equation according to the baseline serum creatinine (sCr). Baseline sCr was the nadir value obtained after the previous admission in those who had more than one admission within 1 year predating the index admission, or the mean outpatient value as baseline when looking back 180 days before index admission in those without previous admission (Shu et al., 2016).

The study excluded patients who had undergone nephrectomy, renal transplantation, or chronic dialysis before the date of operation. Patients with possible AKI caused by obstruction, glomerulonephritis, vasculitis, hemolytic uremic syndrome, or thrombotic thrombocytopenic purpura in the post-surgery conservative period were also excluded.

The study protocol was approved by the Research Ethics Committee of the NTUH (201807119RIND), and the need for informed consent was waived because of the retrospective nature, and no any identifying patient data could be accessed.

### Comorbidities

To analyze the patients’ comorbid conditions, we calculated the Charlson comorbidity index scores (Charlson et al., 1987) from International Classification of disease, 9^th^ Revision, Clinical Modification (ICD-9-CM) coding and ICD-10 administrative data (Quan et al., 2005).

### Measurement of ACEIs/ARBs Exposure

Patients who did not receive ACEIs or ARBs prior to 90 days before surgery were defined as “drug-naïve.” The use of ACEIs or ARBs in this study was defined as prescription of the medication within 90 days before the index surgery. The doses and days of drug use of ACEIs or ARBs were converted to the number of defined daily doses (DDD) as defined by the World Health Organization (WHO). We assigned captopril 50 mg or losartan 50 mg per day a reference DDD value of 1, and all other ACEIs or ARBs and their doses were assigned a corresponding relative intensity according to the WHO ATC/DDD Index 2020 (WHO Collaborating Center for Drug Stastistics Methodology, 2020).

### Outcomes

Outcomes, including AKI, defined according to the Kidney disease: Improving Global Outcomes (KDIGO) guideline (Kellum et al., 2012), and both 30 days mortality after index operation and long-term all-cause mortality following surgery, were collected for analysis. For the most severe AKI, we evaluated the severity of AKI using the peak sCr and the change in sCr during an AKI event. Peak sCr was defined as the maximal value measured within 10 days of an AKI event (Nadkarni et al., 2017).

### Statistical Analysis

The baseline variables were shown as means ± standard deviations (SDs) for continuous variables, and percentages for categorical variables, respectively, in the three different groups. The differences between these groups were compared using the two-tailed unpaired *t*-test for continuous variables and the χ^2^ test for categorical variables, respectively. We compared the risk for AKI and mortality between the above three groups using logistic regression, adjusting for age, gender, medication, comorbidities, and eGFR (Table 1).

**TABLE 1:** Demographic data, comorbidities, medications, and surgery types of the three groups classified by exposure to angiotensin-converting-enzyme inhibitors or angiotensin receptor blockers.

	ACEI users (*n* = 268)	ARB users (*n* = 1,137)	No ACEI/ARB (*n* = 18,871)	*P* value^a^	*P* value^b^	*P* value^c^
Age (years, SD)	59.3 ± 12.3	61.3 ± 10.5	61.4 ± 10.5	0.001	0.128	<0.001
Male gender	180 (67.16%)	707 (62.18%)	9,525 (50.47%)	0.421	<0.001	<0.001
Baseline eGFR (mL/min/1.73 m^2^)	111.4 ± 52.0	99.3 ± 45.8	116.9 ± 50.8	0.948	<0.001	<0.001
Comorbidities						
Chronic kidney disease	27 (10.07%)	161 (14.16%)	828 (4.39%)	0.077	<0.001	<0.001
Liver disease	39 (14.55%)	136 (11.96%)	2,426 (12.86%)	0.248	0.410	0.381
Paralysis	16 (5.97%)	62 (5.45%)	302 (1.60%)	0.739	<0.001	<0.001
Hypertension	242 (90.30%)	1,085 (95.43%)	11,990 (63.54%)	0.001	<0.001	<0.001
Chronic pulmonary disease	26 (9.70%)	120 (10.55%)	1,022 (5.42%)	0.681	0.002	<0.001
Peripheral vascular disorders	5 (1.87%)	62 (5.45%)	186 (0.99%)	0.013	0.198	<0.001
Cardiac arrhythmias	82 (30.60%)	302 (26.56%)	2,051 (10.87%)	0.182	<0.001	<0.001
Congestive heart failure	106 (39.55%)	487 (42.83%)	1,536 (8.14%)	0.328	<0.001	<0.0001
Valvular disease	63 (23.51%)	326 (28.67%)	1,156 (6.13%)	0.089	<0.001	<0.001
Diabetes	110 (41.04%)	460 (40.46%)	4,466 (23.67%)	0.860	<0.001	<0.001
Charlson comorbidity score	2.16 ± 1.27	2.39 ± 1.23	1.39 ± 1.19	0.544	0.130	0.095
Medication						
Alpha-blocker	115 (42.91%)	386 (35.28%)	1,294 (6.86%)	0.042	<0.001	<0.001
Beta-blocker	161 (60.07%)	742 (67.82%)	2,654 (14.06%)	0.011	<0.001	<0.001
Calcium-channel blocker	175 (65.30%)	774 (70.75%)	3,547 (18.80%)	0.042	<0.001	<0.001
COX2 inhibitor	34 (12.69%)	141 (12.89%)	4,621 (24.49%)	0.946	<0.001	<0.001
COX1 inhibitor	42 (15.67%)	156 (14.26%)	4,215 (22.34%)	0.711	0.009	<0.001
Types of major surgery						
Cardiothoracic	185 (69.03%)	857 (75.37%)	7,895 (41.84%)	0.033	<0.001	<0.001
Esophagus	23 (8.58%)	55 (4.84%)	1,731 (9.17%)	0.016	0.739	<0.001
Intestine	38 (14.18%)	111 (9.76%)	3,329 (17.64%)	0.035	0.139	0.601
Liver	31 (11.57%)	170 (14.95%)	6,677 (35.38%)	0.155	<0.001	<0.001
Outcomes						
Acute kidney injury	113 (42.16%)	412 (36.24%)	3,228 (17.11%)	0.071	<0.001	<0.001
Stage 1	94 (35.07%)	351 (30.87%)	2,391 (12.67%)			
Stage 2	8 (2.99%)	17 (1.50%)	367 (1.94%)			
Stage 3	11 (4.1%)	44 (3.87%)	470 (2.49%)			
30-days mortality after index operation	8 (2.98%)	23 (2.02%)	321 (1.70%)	0.335	0.146	0.418
Long-term all-cause mortality	22 (8.21%)	97 (8.53%)	1,680 (8.90%)	0.865	0.692	0.669

a ACEI Users compared to ARB Users

b ACEI Users compared to No ACEI/ARB

c ARB Users compared to No ACEI/ARB

Abbreviation: COX: cyclooxygenase; eGFR: estimated glomerular filtration rate; The stages of AKI were defined by the Kidney disease: Improving Global Outcomes (KDIGO) classification.

Because ACEI/ARB may be a time-dependent property associated with the onset of AKI, we conducted a nested case-control analysis to estimate the odds ratios (ORs) for AKI, comparing with each ACEIs or ARBs user with a non-user. The nested case-control approach has been described as a method that simplifies the cohort analysis when “exposures vary over time and leads to valid estimates of rate ratios with a negligible loss in precision” (Shih et al., 2015; Suissa, 2015). The significance levels for entry (SLE) and for stay (SLS) were set conservatively to 0.15. Then, with the aid of substantive knowledge, the best candidate final logistic regression model was identified manually by dropping the covariates with *p* values >0.05 one at a time until all regression coefficients were significantly different from 0. All the possible factors, including demographic data, baseline eGFR (estimated glomerular filtration rate), comorbidities, and medications, were initially selected as possible predictors to find out the best final logistic regression model. To evaluate the relationship between dosages of ACEIs or ARBs and the odds ratio of postoperative AKI, we adopted a generalized additive model (GAM) with adjustment for the factors listed in Table 1. A forest plot was constructed for the odds ratio of ACEI or ARB use (vs. nonuse) for subsequent AKI according to prior comorbidities and clinical conditions.

Finally, to assess whether the observed associations between ACEI or ARB use and the end points of interest were attributable to differences in health status, we further compared the risk of gastrointestinal bleeding, an outcome believed not to be affected by ACEIs or ARBs use as a specificity test.

All analyses were performed using SAS 9.2 (SAS Institute Inc.) and R software, version 3.4.2 (Free Software Foundation, Inc., Boston, MA) for data analysis and figure plotting. A two-sided *p* value <0.05 was considered to be statistically significant.

## Results

### Patients’ Clinical Characteristics

**FIGURE 1:**
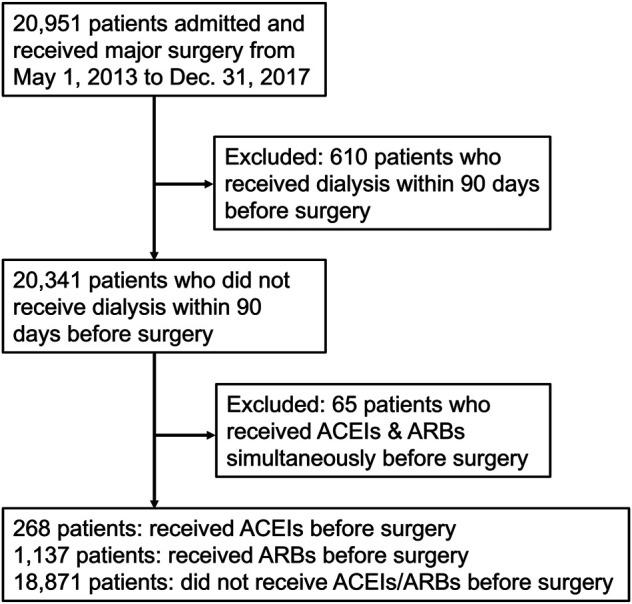
Flow diagram of enrollees. Abbreviations: ACEIs: angiotensin-converting-enzyme inhibitors; ARBs: angiotensin receptor blockers.

The study enrolled 20,951 patients who received major surgery in the study period, of whom 610 patients receiving dialysis and 65 patients receiving ACEIs and ARBs simultaneously within 90 days before surgery were excluded. The remaining 20,276 patients were divided into three groups: those who received ACEIs (*n* = 268), those who received ARBs (*n* = 1,137), and those who received neither ACEIs nor ARBs (*n* = 18,871) within 90 days before surgery (Figure 1).

The clinical characteristics of enrolled patients are shown in Table 1. The use of RAS inhibitors in preoperative patients was mostly attributed to hypertension (*n* = 1,327, 94.4%), congestive heart failure (*n* = 595, 42.2%), followed by diabetes (*n* = 570, 40.6%). A higher proportion of patients who used ACEIs or ARBs before surgery were male (63.13 vs. 50.47%, *p* < 0.001) compared with those who did not. Compared with patients who did not use ACEIs or ARBs, the levels of eGFR were significantly lower in both ACEI users and ARB users (111.4 ± 52.0 and 99.3 ± 45.8 vs. 116.9 ± 50.8 ml/min/1.73 m^2^, respectively, both *p* < 0.001). Regarding the distribution of comorbidities, compared with patients who did not use ACEIs or ARBs, those who received ACEIs or ARBs had significantly higher ratios of chronic kidney disease, hypertension, cardiac arrhythmias, congestive heart failure, valvular heart disease, diabetes, paralysis, and chronic pulmonary disease. However, the Charlson comorbidity scores were not significantly different between these three groups.

Patients who received ACEIs or ARBs also had a much higher ratios of taking other anti-hypertensive agents, including alpha-blockers, beta-blockers, and calcium channel blockers. On the other hand, patients who received ACEIs or ARBs had a significantly lower ratios of taking both cyclooxygenase (COX) 1 and COX2 inhibitors. Compared with patients without ACEIs or ARBs, a higher proportion of ACEI and ARB users underwent cardiac surgery (69.03 and 75.37% vs. 41.84% respectively, both *p* < 0.001).

### Risk for Post-operative AKI in Patients Who Received ACEIs, ARBs, and Neither ACEI nor ARB

In our study, of a total of 20,276 enrolled patients, there were 3,753 postoperative AKI events overall (18.51%), mostly KDIGO stage 1 AKI (2,836 patients, 75.6% cases of total AKI), whereas overall mortality following surgery was 1,799 patients (8.87%).

**FIGURE 2:**
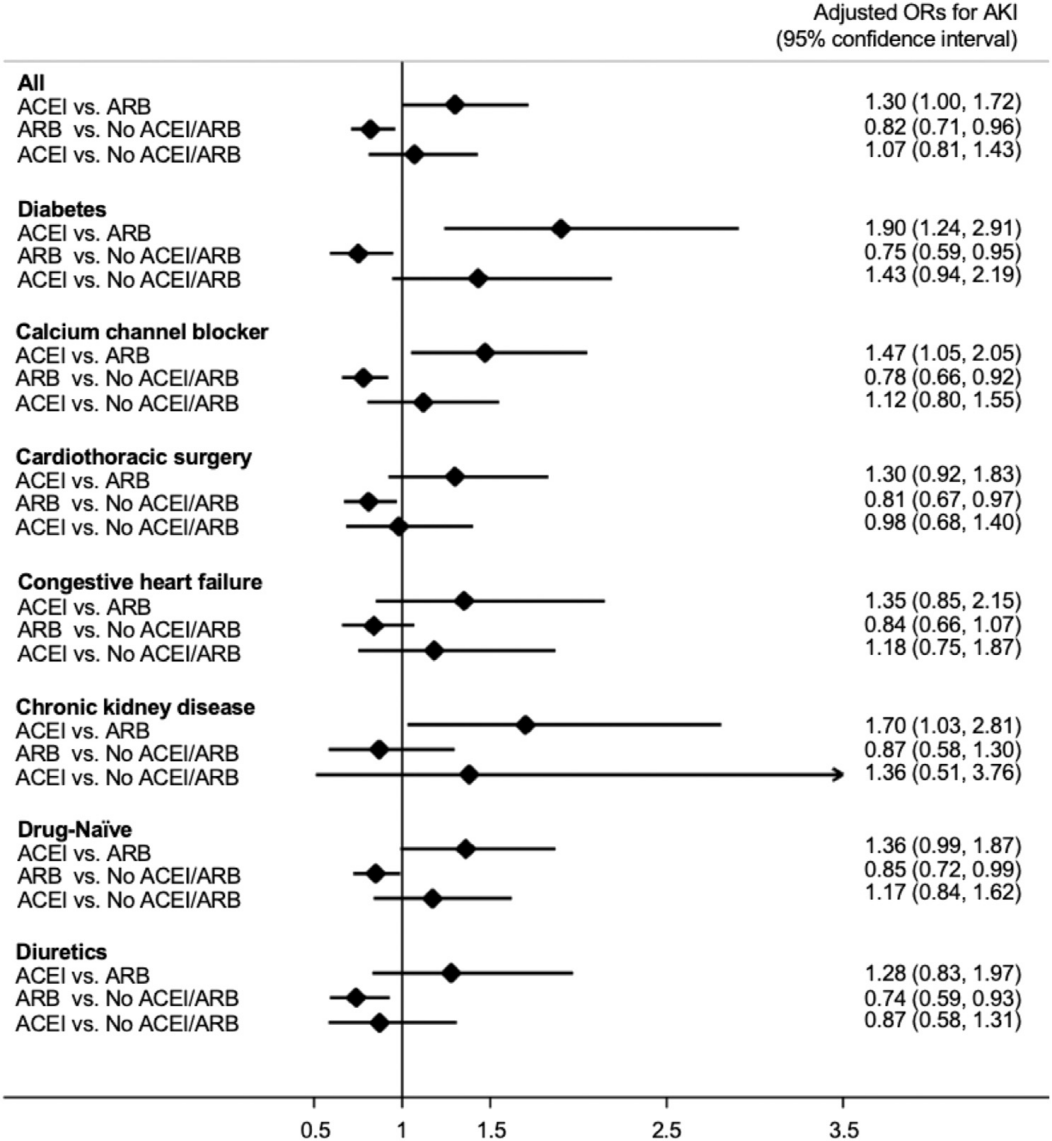
Forest plot demonstrating the adjusted odd ratios for postoperative acute kidney injury in different subgroups with comparison between various conditions of ACEI or ARB usage. Abbreviations: ACEI: angiotensin-converting-enzyme inhibitor; ARB: angiotensin receptor blocker; No ACEI/ARB: No ACEI or ARB use within 90 days before surgery; Drug-Naïve: No ACEI or ARB use prior to 90 days before surgery.

As shown in Figure 2, patients who received ACEIs had a higher risk for postoperative AKI than those who received ARBs (adjusted odds ratio (OR); 95% confidence interval (CI): 1.30; 1.00–1.72) by multivariable logistic analysis. However, compared with patients without ACEI/ARB, patients who received ARBs had a significantly lower risk for postoperative AKI (adjusted OR; 95% CI: 0.82; 0.71–0.96). The risk for postoperative AKI was not significantly different between the patients who received ACEIs and those who did not receive ACEI/ARB (adjusted OR; 95% CI: 1.07; 0.81–1.43).

We also acknowledged similar findings in the subgroup analysis (Figure 2). In patients with diabetes, those who received calcium channel blockers or diuretics, those who received cardiac surgery, and “drug-naïve” patients, the risk for postoperative AKI was significantly lower in those taking ARBs compared with those taking neither ACEIs nor ARBs. In addition, in patients who received calcium channel blockers and those with diabetes or chronic kidney disease the use of ACEIs is associated with a significantly increased risk for postoperative AKI compared with the use of ARBs.

### Risk for Long-Term All-Cause Mortality Following Surgery in Patients Who Received ACEIs, ARBs, and Neither ACEIs nor ARBs

**FIGURE 3:**
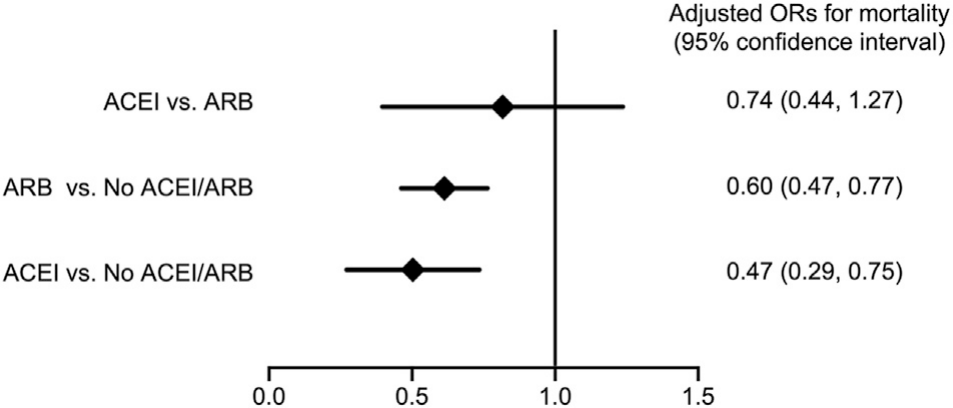
Forest plot depicts the adjusted odd ratios for long-term all-cause mortality following major surgery with comparison between various conditions of ACEI or ARB usage. Abbreviations: ACEI: angiotensin-converting-enzyme inhibitor; ARB: angiotensin receptor blocker; No ACEI/ARB: No ACEI or ARB use within 90 days before surgery.

As shown in Figure 3, compared with patients without ACEI/ARB, both patients who received ACEIs and ARBs had a significantly reduced risk for long-term (average, 380 ± 402 days) all-cause mortality following major surgery (adjusted OR; 95% CI: 0.47; 0.29–0.75 in ACEI users, whereas adjusted OR; 95% CI: 0.60; 0.47–0.77 in ARB users) after adjusting for age, gender, and eGFR. There was no significant difference in the risk for long-term all-cause mortality following surgery between patients who received ACEIs and ARBs (adjusted OR; 95% CI: 0.74; 0.44–1.27). In addition, comparing with the non-users, there were no significant differences on 30-days mortality after index operation in both ACEI and ARB users (adjusted OR; 95% CI: 1.49; 0.76–2.22 in ACEI users and 0.97; 0.34–1.61 in ARB users). The 30-days mortality did not differ significantly between ACEI and ARB users (adjusted OR; 95% CI: 1.53; 0.85–2.20).

### Analysis of the Effect of ACEI or ARB Dosage on the Probability of AKI

**FIGURE 4:**
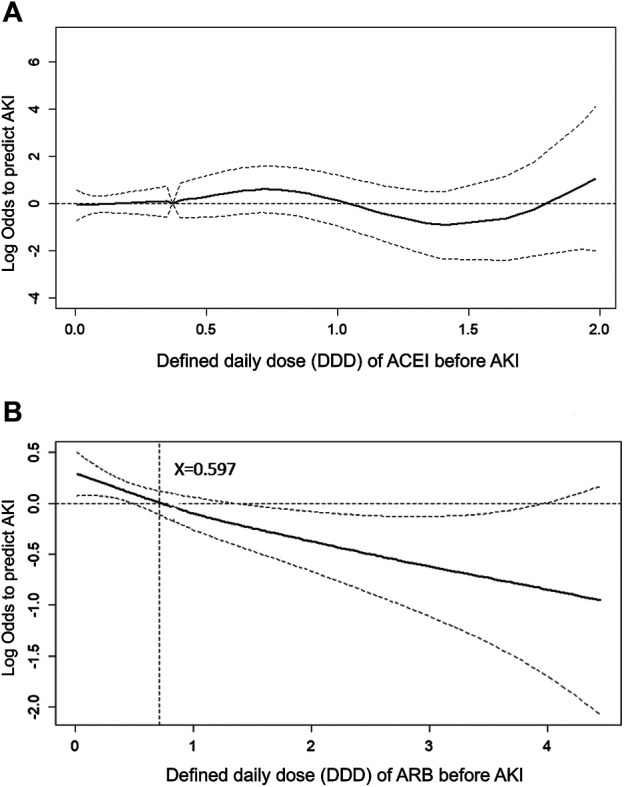
Generalized additive model plot of the probability of postoperative acute kidney injury for ACEI or ARB usage quantity. The probability of postoperative AKI, expressed as the logarithm of the odds, was constructed with the defined daily dose of ACEI **(A)** or ARB **(B)** usage and was centered to have an average of zero over the range of the data as constructed with the GAM. Both models are adjusted for age; gender; baseline eGFR; and the use of cyclooxygenase inhibitors, alpha-blockers, and calcium channel blockers.

To evaluate the effect of ACEI or ARB dosage on the probability of postoperative AKI in patients who received major surgery, we adopted a generalized additive model (GAM) with adjustment for age, gender, baseline eGFR, and concomitant medications (listed in Table 1). The results are shown as a function curve with the dosages of ACEIs or ARBs, represented as DDD, against the values of the logs of odds ratios for postoperative AKI (Figures 4A,B). This approach permits adjustments for possible nonlinear effects of continuous variables (Uchino et al., 2004; Burden et al., 2017). The use of ARBs was found to reduce the probability of postoperative AKI in a dose-dependent manner (Figure 4B), whereas the use of ACEIs did not change the probability of postoperative AKI (Figure 4A). Even a few DDDs of ARB treatment in patients, e.g., 0.597, attenuated the postoperative risk of AKI, and this protective effect was consistent.

### Complication Analysis

We further analyzed the episodes of hyperkalemia, defined as serum potassium greater than 5.3 mmol/L (upper limit of the normal range), and found that the use of ACEIs or ARBs did not increase the risk of hyperkalemia during the index hospitalization (*p* = 0.199).

### Specificity Analysis

To attribute the possible health indication biases or unobserved confounders, we identified the risk of new onset of gastrointestinal bleeding. Patients who used ACEIs or ARBs had a similar risk of gastrointestinal bleeding during the index hospitalization (*p* = 0.242).

## Discussion

The present nested case-control study revealed that patients who received ARBs had a reduced risk of postoperative AKI after major surgery, but those who received ACEIs did not. We also found that both patients who received ARBs and those received ACEIs had a reduced risk of long-term mortality following surgery, whereas the risk of hyperkalemia did not increase. ARBs were also found to reduce the probability of postoperative AKI in a dose-dependent manner.

### ACEI and ARB Differ in Their Clinical Scenarios and Basic Mechanisms

Both ACEIs and ARBs are RAS inhibitors, and they had been considered to have similar clinical effects. However, our present study provides evidence that their effects on reducing the risk of AKI after major surgery may be different. Many previous studies classified ACEIs and ARBs in the same group and thus may have failed to address their respective beneficial and adverse effects. Separate exploration for the effects of ACEIs and ARBs may help to elucidate their respective effects on important outcomes in various clinical scenarios. In treating patients with hypertension, heart failure, diabetes, cardiovascular disease, and chronic kidney disease, head-to-head studies revealed that the anti-hypertensive efficacy and various clinical outcomes between ACEIs and ARBs were comparable, though the withdrawal rates resulting from adverse events in ARB users were reported to be lower (Messerli et al., 2018). However, in some clinical scenarios, ARBs provided better protection than ACEIs. For example, in the REACH cohort, ARBs were superior to ACEIs at reducing CV events in high-risk patients in real-world practice (Potier et al., 2017), whereas in patients who received coronary artery bypass grafts, the incidence of major CV events was significantly lower in ARB users during the 12 months follow-up period by propensity-matched analysis (Kim et al., 2020). In addition to CV protection, ARBs were associated with lower rates of sepsis than ACEIs in patients with chronic obstructive pulmonary disease (Lai et al., 2019), whereas patients who used ARBs but not ACEIs had lower rates of hospitalization for sepsis than untreated patients (Dial et al., 2014).

In patients who received ARBs or ACEIs, plasma angiotensin II was augmented in the ARB group but not in the ACEI group (Nakamura et al., 2009); thus, in patients who receive ARBs, increased levels of angiotensin II may facilitate angiotensin II type 2 receptor (AT2R)-mediated effects because the angiotensin II type 1 receptors are blocked by these drugs. AT2R-mediated beneficial effects, including natriuresis, vasodilation, anti-inflammation, and anti-oxidation, may provide CV and renal protective effects (Chow and Allen, 2016). In addition to AT2R-mediated effects, ARBs could also ameliorate renal injury through the angiotensin-converting enzyme 2 (ACE2)/Mas axis (Callera et al., 2016). ACE2, expressed broadly in the kidney, is a key counter-regulatory enzyme that degrades angiotensin II to angiotensin-(1–7), thereby attenuating the effects of angiotensin II on vasoconstriction, sodium retention, and fibrosis (Williams and Scholey, 2018). ARBs increase renal ACE2 expression (Soler et al., 2009; Chow and Allen, 2016); however, ACEIs in clinical use do not directly affect the activity of ACE2 (Rice et al., 2004). Increased expression of ACE2 and plasma angiotensin-(1–7) in animals and patients are found after using ARBs; therefore, ARBs could play a pivotal role by modifying processes associated with acute inflammation. This effect could be via the ACE2/Ang-(1–7)/Mas axis, such as reducing oxidative stress and inhibiting leukocyte recruitment and activation (Simões e Silva et al., 2013).

The differences between ARBs and ACEIs observed in our study may result from the effects of ARB in vasodilation an anti-inflammation via AT2R and the ACE2/Mas receptor pathways mentioned above. These mechanisms supported that ARBs may remedy the kidney outcomes and reduce mortality after major surgery, in which renal and systemic hypoperfusion and inflammation are common (Gumbert et al., 2020).

### ARBs, but Not ACEIs, Decreased Postoperative AKI

Major surgery is the frequently met clinical scenario, thus it is an important issue to explore the effects of preoperative interventions of ACEIs or ARBs on postoperative AKI and mortality after major surgery because these therapeutic interventions may influence postoperative outcomes. Renal and systemic hypoperfusion and inflammation are common in major surgery and associated with perioperative AKI and mortality.

Our findings from the present real-world cohort, using nested case-control analysis modeling, has confirmed that ARB use is associated with a lower probability of subsequent AKI after major surgery, but ACEIs use is not. Furthermore, a similar protective effect was also noted in subgroup analysis of new (ACEI/ARB-naïve) ARBs users. In light of our findings, both new use of ARB in ACEI/ARB-naïve patients or just maintaining ARB use before a scheduled major surgery may be helpful.

In addition, as shown by the GAM plot in Figure 4, ARBs reduced the risk of AKI in a dose-dependent manner; however, a dose-response relation was not found in ACEIs. The dose-response relation of ARBs against AKI after major surgery provided evidence supporting causality between ARBs and reduced postoperative AKI, though large-scale, randomized controlled trials are required to confirm this.

### Both ACEI and ARB Decreased Postoperative Long-Term All-Cause Mortality

Beyond the effects on postoperative AKI, we also found that preoperative use of both ACEIs and ARBs within 90 days before surgery were associated with reduced postoperative long-term all-cause mortality. Our findings are novel and clinically important, supporting preoperative use of ACEIs/ARBs, at least within 90 days before major surgery. A large previous population-based retrospective cohort study enrolling 237,208 patients aged 66 years or older (Shah et al., 2014) revealed that preoperative ACEI/ARB use vs. non-use within 120 days prior to major elective surgery was associated with a 9% lower risk of all-cause mortality within 90 days of surgery. Compared with this study, which only enrolled elderly patients (mean age 74 years), the present study (mean age 61 years) revealed a much better reduction of mortality risk (40% reduction in ARB users and 53% reduction in ACEI users). We also showed that the 30 days mortality after index operation, a possibly estimated mortality attributed directly to operation, did not differ statistically in the three groups. The discrepancy between 30 days mortality after index operation and long-term all-cause mortality may help to support that ACEIs and ARBs improved long-term outcome after major surgery, without influencing the mortality attributed directly to operation.

### Study Strengths and Limitations

Our study has some limitations. First, it was an observational study; therefore, the associations were not prospective, and causality cannot be inferred. The observational nature of this study was an intrinsic limitation because the lack of randomization precluded a definite investigation of treatment advantages. However, it is very difficult to perform a randomized controlled trial because ACEIs and ARBs are strongly recommended therapeutic agents in treating hypertensive patients with chronic kidney disease or congestive heart failure. Second, the ratios of postoperative AKI (18.51%) and mortality (8.87%) in our study are high relative to those in previous studies, possibly due to the severity of major surgery and possibly higher ratio of shock (though the actual ratio of shock was unknown) in our study. Third, we have highlighted the importance of ARBs in surgery-related AKI and designed a nested case-control study. It was recently asserted that a restriction should be placed on control selection in nested case-control studies to make cases and controls more comparable and minimize selection as well as introduce bias (Sedgwick, 2010). Fourth, obviously, the treating physicians had already performed a risk assessment and decided that the benefits of ACEIs/ARBs outweighed the potential nephrotoxicity. However, the disease severity scores were similar between the study groups. Indeed, the ratios of comorbidities of diabetes and congestive heart failure were even more frequent in ARB users, while ARB users had less surgery-related AKI. Moreover, using the validated outcome of gastrointestinal bleeding that was not interfered by ACEIs/ARBs, we could confirm that selection bias was not significant from our study design. Additionally, we have further balanced the risks by multivariable regression analysis and showed coherent results.

## Conclusion

In conclusion, the present study revealed that preoperative use of ARBs was associated with a reduced risk of postoperative AKI, in a dose-dependent manner. Preoperative use of either ACEIs or ARBs was associated with reduced long-term mortality following major surgery, whereas the risk of hyperkalemia was not increased. These results revealed that preoperative ARB use helps to reduce postoperative AKI and long-term mortality. Further prospective randomized studies are needed to verify our findings.

## Data Availability

The raw data supporting the conclusion of this article will be made available by the authors, without undue reservation.
